# One-step PCR: A novel protocol for determination of *pfhrp2* deletion status in *Plasmodium falciparum*

**DOI:** 10.1371/journal.pone.0236369

**Published:** 2020-07-23

**Authors:** Sophie Jones, Gireesh Subramaniam, Mateusz M. Plucinski, Dhruviben Patel, Jasmine Padilla, Michael Aidoo, Eldin Talundzic

**Affiliations:** 1 Malaria Branch, Division of Parasitic Diseases and Malaria, Center for Global Health, Centers for Disease Control and Prevention, Atlanta, Georgia, United States of America; 2 Williams Consulting, Baltimore, Maryland, United States of America; 3 Oak Ridge Institute for Science and Education, Atlanta, Georgia, United States of America; 4 President’s Malaria Initiative, Atlanta, Georgia, United States of America; 5 Biotechnology Core Facilities Branch, Division of Scientific Resources, Centers for Disease Control and Prevention, Atlanta, Georgia, United States of America; Instituto Rene Rachou, BRAZIL

## Abstract

Histidine-rich protein 2 (HRP2) detecting rapid diagnostic tests (RDTs) have played an important role in enabling prompt malaria diagnosis in remote locations. However, emergence of *pfhrp2* deleted parasites is threatening the efficacy of RDTs, and the World Health Organization (WHO) has highlighted surveillance of these deletions as a priority. Nested PCR is used to confirm *pfhrp2* deletion but is costly and laborious. Due to spurious amplification of paralogue *pfhrp3*, the identity of nested exon 1 PCR product must be confirmed by sequencing. Here we describe a new one-step PCR method for detection of *pfhrp2*. To determine sensitivity and specificity, all PCRs were performed in triplicate. Using photo-induced electron transfer (PET) PCR detecting 18srRNA as true positive, one-step had comparable sensitivity of 95.0% (88.7–98.4%) to nested exon 1, 99.0% (94.6–99.9%) and nested exon 2, 98.0% (93.0–99.8%), and comparable specificity 93.8% (69.8–99.8%) to nested exon 1 100.0% (79.4–100.0%) and nested exon 2, 100.0% (74.4–100.0%). Sequencing revealed that one step PCR does not amplify *pfhrp3*. Logistic regression models applied to measure the 95% level of detection of the one-step PCR in clinical isolates provided estimates of 133p/μL (95% confidence interval (CI): 3-793p/μL) for whole blood (WB) samples and 385p/μL (95% CI: 31–2133 p/μL) for dried blood spots (DBSs). When considering protocol attributes, the one-step PCR is less expensive, faster and more suitable for high throughput. In summary, we have developed a more accurate PCR method that may be ideal for the application of the WHO protocol for investigating *pfhrp2* deletions in symptomatic individuals presenting to health care facilities.

## Introduction

Malaria remains a serious public health threat that was responsible for 405,000 deaths in 2018 [1]. While impressive gains have been made in reducing associated morbidity and mortality, progress has slowed for multiple reasons, and in the 10 highest burden countries there were 3.5 million more cases in 2017 than had been reported for 2016 [2]. Rapid and accurate diagnosis and prompt deployment of effective antimalarial medication are essential components of appropriate case management. Currently, diagnosis in remote resource limited locations is possible through use of antigen detecting rapid diagnostic tests (RDTs) which are inexpensive, easy to use, provide a result in <30 minutes and do not require special equipment or an electricity source [3]. This appealing combination of attributes, along with the high sensitivity and specificity observed among some products [4], has firmly cemented RDTs at the heart of malaria diagnosis, where they have played a central role in contributing to malaria control since their large-scale introduction in the early 2010s.

Three *Plasmodium* parasite antigens, histidine rich protein 2 (HRP2)(*Plasmodium falciparum* detection only), plasmodium lactate dehydrogenase (pLDH), and aldolase, have been used as diagnostic targets in RDTs [5]. Of these, HRP2-specific tests offer the most sensitive detection of *P*. *falciparum* and are also comparatively less susceptible to degradation by heat and humidity during storage [4]. Consequently, the vast majority of malaria RDTs procured and distributed have an HRP2 detecting band [4].

Reports of false negative RDT results in 2010 led to the discovery that a substantial proportion of *P*. *falciparum* parasites from Peru had part or the entire *pfhrp2* gene deleted [6], threatening to compromise the suitability of this valuable tool. Concurrently, deletion of the gene encoding the paralogue *P*. *falciparum* histidine rich protein 3 (*pfhrp3*), which cross reacts with HRP2-detecting RDTs at high parasitaemias due to sequence similarities [7] was discovered in the same region at a higher prevalence [6]. Since this initial discovery in Peru and neighboring countries [8–10], reports of *pfhrp2* deleted parasites emerged from India [11] followed by suspected low prevalence deletion in Mali [12], Senegal [13], Yemen [14], Bangladesh [15], Myanmar [16], Zambia [17], Ghana [18], Democratic Republic of Congo [19], Uganda [20], Rwanda [21], Kenya [22], Mozambique [23], Angola [24], Nigeria [25] and Equatorial Guinea [26]. In Eritrea, particularly high *pfhrp*2 deletion prevalences of 41.7% and 80.8% were reported from two hospitals [27]. In the African continent, the spread of *pfhrp2* deleted parasites presents a particularly severe public health threat, since disease burden is high and HRP2-only detecting RDTs have been widely deployed in most countries due to predominance of falciparum only malaria [27]. The concern is that *pfhrp2* deleted parasites may go undetected by HRP2 detecting RDTs and infections may remain undiagnosed and untreated, therefore worsening morbidity and mortality. Additionally, untreated *pfhrp2* deleted parasites would continue to be transmitted further, thus propagating the problem. Since no other current tool offers rapid, sensitive detection of *falciparum* malaria, there is an urgent need to preserve the HRP2-detecting RDT, or identify suitable alternative targets.The World Health Organization (WHO) recommends switching to the slightly less sensitive pLDH-detecting products if *pfhrp2* deletion prevalence reaches ≥5% [28]. The development of molecular surveillance tools to track the frequency and distribution of *pfhrp2*-deleted parasites has become a top global health priority. In the laboratory, proving absence of a gene is complex, since negative PCR results can occur for other reasons, such as low parasitaemia, low quality DNA or an insensitive PCR assay [29]. Additionally, laboratory amplification of *pfhrp2* is complicated by the high genetic variability within the gene, which hampers efforts to locate conserved regions to design PCR primers. *Pfhrp2* is located on chromosome 8 in the sub-telomeric region which is a dynamic area subject to frequent genetic recombination, and at risk of chromosomal breakage [15, 30–32]. Consequently, HRP2 has a very complex structure, and also has varying numbers of histidine-alanine rich repeat motifs, which are classified into at least 29 repeat types that vary in size and frequency among strains [7, 33, 34]. Repeats type 2 and 7 are thought to be the target for RDT monoclonal antibodies and since both can appear in HRP3, cross reactivity of HRP3 is observed in HRP2 detecting RDTs [33].

The WHO recommends that *pfhrp2* deletion be explored in symptomatic individuals presenting to a healthcare facility whose infection fits the following criteria: microscopy positive (especially high density infections), HRP2 RDT negative and ideally Pf-pLDH RDT positive [28]. To provide evidence of absence of *pfhrp2*, 2 single copy genes also need to be amplified to eliminate poor quality DNA as the cause of the negative *pfhrp2* PCR result. Therefore, *pfhrp2* deletion is identified as a negative result for *pfhrp2* PCR, but positive result for single copy gene PCR reactions. However, it is important to be aware of the limitations of each PCR assay to ensure samples are not deemed negative when they simply have low parasitaemia close to the detection limit of any of the assays used [35].

The aim of this study was to develop a faster, simpler, more specific PCR assay for amplification of the whole of *pfhrp2* in one-step.

## Materials and methods

### Study design

Assessment of the current gold standard nested PCR [36] and our new one-step PCR method was performed using three sample sets. The first set, comprising 7 well-characterized reference samples cultured at the US Centers for Disease Control and Prevention (CDC), Atlanta, was used to confirm PCR assay specificity. *P*. *falciparum* isolates 3D7 (suspected origin of Africa), 7G8 (Brazil), HB3 (Honduras) and D6 (Sierra Leone), all three of which express *pfhrp2* were positive controls, whereas Dd2 (Indo-china), D10 (Papua New Guinea) and 3BD5 (lab cross of Dd2 and HB3), which lack *pfhrp2* were negative controls, along with malaria free donor blood from Interstate Blood Bank, Tennessee. All samples contained *pfhrp3* except HB3 and 3BD5.

The second sample set was used to determine the analytical sensitivity of each method. Five different culture adapted isolates, originating from Nigeria, El Salvador, The Philippines, Benin and Papua New Guinea that encompass HRP2 types A, B and C, were titrated in ten-fold dilutions from 200p/μL to 0.002p/μL in nuclease free water (Thermofisher Scientific, MA, USA). Each dilution was tested against each assay in triplicate.

The third set comprised anonymized clinical samples collected from the following four sources: the CDC’s US domestic malaria surveillance unit (n = 106, predominantly from West Africa), a therapeutic efficacy study in Angola (n = 10) [37], a health facility survey in Mozambique (n = 24) [38] and 20 Peruvian samples collected for the specimen bank used for the Malaria RDT product testing programme. The US domestic malaria surveillance samples were suspected imported cases of malaria, mostly from travelers seeking medical treatment at a US healthcare facility. These samples were sent to the CDC reference malaria diagnostic lab for confirmation of parasite status and 91 samples were found to be photo-induced electron transfer (PET) PCR positive for *P*. *falciparum* and 16 samples were negative for all species. The Angolan samples were taken from symptomatic children with microscopically confirmed *P*. *falciparum* infection, whereas the Mozambican samples were taken from febrile individuals with positive RDT results spanning all ages. It is important to note, these sample sizes are small. These samples were used to determine the sensitivity, specificity, positive predictive value and negative predictive value of the PCR methods against patient isolates. Each clinical isolate was tested in triplicate against each of the PCR methods, with any positive replicate deeming that sample detected. This study was reviewed and approved by CDC internal review clearance process.

### DNA extraction

DNA was extracted from whole blood (WB) for the reference samples, cultured isolates, domestic surveillance samples and Peruvian isolates, and from dried blood spots (DBS) for the Angolan and Mozambican samples. The QIAamp DNA Mini Kit (Qiagen, Hilden, Germany) was used, following the ‘DNA Purification from Blood’ or ‘DNA purification from Dried Blood Spots” protocols according to the manufacturer recommendations. The same volume of WB was used for all WB samples, according to protocol. Following both protocols, DNA was eluted in nuclease free water, then stored at -20°C until use.

PET PCR was performed to confirm parasite presence and allow an estimate of density in all samples. The protocol amplifies 18srRNA, has a reported limit of detection (LOD) of 3.2p/μL for *P*. *falciparum* detection and was performed following protocols described elsewhere [39] for all samples, excluding the domestic surveillance samples which were assayed outside of this study.

### PCR amplification of *pfhrp2*

#### Nested PCR

Nested PCR was performed as detailed elsewhere [36, 40]. Briefly, 20μL reactions were assembled with the following end concentrations: 0.75μM forward primer, 0.75μM reverse primer, 1 x Expand High Fidelity reaction buffer (Roche, Basel, Switzerland), 200μM dNTPs (New England Biolabs, MA, USA), 0.7U/μL Expand High Fidelity Enzyme mix (Roche, Basel, Switzerland), and 2μL of DNA template. Mastermix preparations were the same for nest 1 and nest 2 PCRs for both exon 1 and 2.

For preparation for use in the nest 2 PCR reaction, the exon 1 nest 1 PCR product was first diluted 1:10 in nuclease free water, then 2μL of diluted sample used as template in the nest 2 reaction. The nest 2 PCR product (~222 base pairs) was then visualized on a 2.0% TBE (Tris-Borate-EDTA) gel. For exon 2, the nest 1 product was diluted 1:200 in nuclease free water, and 2μL served as template for the nest 2 PCR. The exon 2 product (~748 base pairs), was visualized on a 1.5% TBE gel. Table 1 details all primer names, sequences and PCR cycling conditions.

**Table 1:** PCR primer sequences and thermocycling conditions.

gene	PCR method	Primer name	Sequence (5’-3’)	Thermocycling conditions
*pfhrp2*	Exon 1- nest 1, forward	2E12F1	GGT TTC CTT CTC AAA AAA TAA AG	95°C for 5 min, (95°C for 30 s, 55°C for 30 s, 68°C for 30 s) x 30 cycles, 68°C for 5 min.
	Exon 1- nest 1, reverse	2E12R1	TCT ACA TGT GCT TGA GTT TCG	
	Exon 1- nest 2, forward	2E12F	GTA TTA TCC GCT GCC GTT TTT GCG	95°C for 5 min, (95°C for 30 s, 62°C for 30 s, 68°C for 30 s) x 30 cycles, 68°C for 5 min.
	Exon 1- nest 2, reverse	2E12R	CTA CAC AAG TTA TTA TTA AAT GCG GAA	
	Exon 2- nest 1, forward	2E2F	TTC CGC ATT TAA TAA TAA CTT GTG	95°C for 5 min, (95°C for 30 s, 55°C for 30 s, 68°C for 1 min) x 30 cycles, 68°C for 5 min.
	Exon 2- nest 1, reverse	2E2R1	GGC AAT GTG TGG CGG CTT C	
	Exon 2- nest 2, forward	2E2F1	CGA AAC TCA AGC ACA TGT AGA	95°C for 5 min, (95°C for 30 s, 57°C for 30 s, 68°C for 1 min) x 20 cycles, 68°C for 5 min.
	Exon 2- nest 2, reverse	2E2R	CTT CGT GGT GTG CGG CTG	
	One-step, forward	BRAVO_F	ATG ATT CAT TAT TCT ATA TTT ATA AGG AAG ATT AC	98°C for 3 min, (98°C for 30 s, 60°C for 1 min 30 seconds, 68°C for 2 mins) x 30 cycles, 68°C for 5 min.
	One-step, reverse	BRAVO_R	CAC TTC ATG TAT TTA TGT ATG CAG AAC	
*pfmsp-1*	Nest 1 & nest 2, forward	M1-OF	CTA GAA GCT TTA GAA GAT GCA GTA TTGATT CTA ATT CAA GTG GAT CAG	95°C for 5 min, (95°C for 30 s, 51°C for 30 s, 68°C for 1 min) x 30 cycles, 68°C for 5 min.
	Nest 1, reverse	M1-OR		
	Nest 2, reverse	M1-IR	CAT ATC CAT CAA TTA AAT ATT TGA AAC C	95°C for 5 min, (95°C for 30 s, 52°C for 30 s, 68°C for 1 min) x 30 cycles, 68°C for 5 min.
*pfmsp-2*	Nest 1, forward	F1	GAA GGT AAT TAA AAC ATT GTC	95°C for 5 min, (95°C for 30 s, 50°C for 30 s, 68°C for 1 min) x 30 cycles, 68°C for 5 min.
	Nest 1, reverse	R1(2)	GAT GTT GCT GCT CCA CAG	
	Nest 2, forward	F2	GAG TAT AAG GAG AAG TAT G	95°C for 5 min, (95°C for 30 s, 48°C for 30 s, 68°C for 1 min) x 30 cycles, 68°C for 5 min.
	Nest 2, reverse	R2	CTA GAA CCA TGA ATA TGT CC	

#### One-step PCR

**Fig 1:**
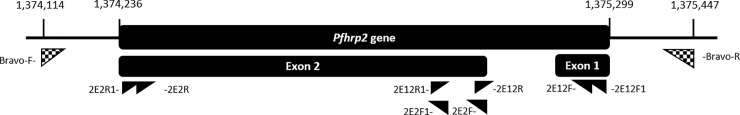
Location of novel one-step primers, and nested *pfhrp2* primers. Locations on chromosome 8, are listed above the schematic. Nested PCR primers are explained in Table 1. One-step primers are shown in a checked pattern. Primer pairing is detailed in Table 1. Note: primers 2E12R1 and 2E2F1 occupy the same location.

To design novel primers, over 1500 *P*. *falciparum* genomes from the Pf3k MalariaGen project (https://www.malariagen.net/apps/pf3k/release_3/index.html, accessed on 05/10/2018) were inspected to identify potential primer binding regions that amplified the full-length of *pfhrp2*. Additional sequences from PlasmoDB (https://www.malariagen.net/apps/pf3k/release_3/index.html) were downloaded and aligned in Geneious Prime (https://www.geneious.com) [41] and regions of low genetic complexity were selected for primer binding sites. The novel primers amplify exon 1 and 2 of *pfhrp2* in one segment, from just outside the gene (location 1,374,114–1,375,447 on chromosome 8, Fig 1).

PCR was set up in 50μL reactions, with the following end concentrations 0.5μM BRAVO-F primer, 0.5μM BRAVO-R primer (see Table 1 for sequences), 1 x Q5 reaction buffer (New England Biolabs, MA, USA), 200μM dNTPs (New England Biolabs, MA, USA), 0.02U/μL Q5 DNA polymerase (New England Biolabs, MA, USA), and 5μL of template DNA. PCR product (~1333 base pairs, depending on repeat region composition) was visualized on a 1.0% TBE gel. See S1 Table for the mastermix preparation table.

#### Application of the WHO *pfhrp2* deletion determination algorithm

Two single copy genes were amplified to enable application of the WHO protocol to identify *pfhrp2* deletion. Merozoite surface protein-1 (*pfmsp-1*) and *pfmsp-2* genes were amplified using nested PCR and the same mastermix and concentrations outlined above for the nested *pfhrp2* PCR protocols, using similar primers to those published previously [42]. The only variation to the *pfhrp2* nested masternix was that 1μL of primary reaction was used as template in the nest 2 reaction, increasing the volume of water by 1μL, for both *pfmsp-1* and *pfmsp-2*. *pfmsp-1* product, approximately 400–550 base pairs, was visualized on a 1.5% TBE gel, and *pfmsp-2* product with an expected size of 500–700 base pairs was also visualized on a 1.5% TBE gel. Primers and thermocycling conditions are outlined in Table 1.

#### Sanger sequencing

To confirm one-step PCR amplicons were *pfhrp2* and not paralogue *pfhrp3*, and to explore *pfhrp2* deletion patterns, a Sanger sequencing protocol was developed. PCR amplicons were prepared as outlined next: 35μL of one-step PCR product was purified using the Monarch PCR and DNA Cleanup Kit (New England Biolabs, MA, USA), according to the manufacturer instructions. DNA concentration was then quantified using the Qubit Fluorometer (ThermoFisher Scientific, MA, USA) and the Qubit dsDNA High Sensitivity kit according to the manufacturer guidelines. The following cycle sequencing reaction was prepared, 6.4μL of ddH_2_0 was combined with 3μL of 4–20μM primer, 3.6μL of BigDye Terminator v1.1 and v3.1 5 x sequencing buffer (ThermoFisher Scientific, MA, USA), 1μL of BigDye (ThermoFisher Scientific, MA, USA), and 6μL of the cleaned one-step PCR amplicon (concentration 40-100ng), with separate reactions performed for the forward and reverse primer. Cycling conditions were as follows, 96°C for 20 seconds, 50°C for 20 seconds and 60°C for 150 seconds for 45 cycles, followed by a hold at 4°C. Product was cleaned using CleanSEQ (Beckman Coulter Inc., CA, USA) according to manufacturer instructions, then sequenced on an ABI 3730XL sequencer (Applied Biosystems, CA, USA).

Raw AB1 sequence traces were analysed using Geneious Prime, trimming regions with more than a 5% chance of error per base, and performing visual assessment of chromatograms to correct discordant basecalling. *De novo* assembly was performed, the consensus sequence was translated and amino acid repeat types identified, labelled and counted.

Pairwise alignments were performed from the consensus sequences, separately comparing amplicon consensus sequence with *pfhrp2* and *pfhrp3* sequences taken from 3D7 reference strain (version 3.0) sourced from PlasmoDB (https://plasmodb.org/plasmo/ accessed on July 2017). Alignment scores and % pairwise identity were used to determine if sequences were *pfhrp2* or *pfhrp3*. To further validate amplicon identity, nucleotides were translated, then amino acid sequences checked for the presence of ‘ANHGFHFNLHDNNSHTLHHAKANACFDD’, which is a highly conserved region that appears at least once in HRP3, but not HRP2 [33]

In addition to traditional assessment of PCR methods, methodological attributes, including hands on time and costs, according to how the protocols were performed at CDC were also assessed.

#### Statistical analysis

Sensitivity, specificity, positive predictive value (PPV) and negative predictive value (NPV) were calculated for both nested PCRs and the one-step PCR in GraphPad Prism version 8.1.2 (GraphPad Software, La Jolla California USA, www.graphpad.com/), using standard calculations. To assess the sensitivity of the one- step PCR, by different parasite densities, and then separately by sample type, non-parametric LOESS curves [43] and logistic regression models [44] were fitted using R Studio version 3.5.0 (R Foundation for Statistical Computing, Vienna, Austria, www.r-project.org/), to estimate the parasite density at which 95% of PCR reactions would be expected to be positive (with 95% confidence intervals).

## Results

### Comparison of PCR methods

#### Cultured isolates- amplicon identity validation

*pfhrp2* was PCR amplified in reference samples using the new one-step method. All isolates known to express *pfhrp2* (3D7, 7G8, HB3 and D6), produced amplicon, whereas those known to be *pfhrp2* deleted (Dd2, D10 and 3BD5, and negative control blood) did not.

*Cultured isolates*: *Analytical sensitivity*. The detection threshold, defined here as the last dilution at which 100% (15/15) of replicates were detected, was identified as 20p/μL for the exon 1 nested and the one-step protocols and >200p/μL for the exon 2 nested protocol (Table 2).

**Table 2:** Analytical sensitivity of PCR methods against titrated cultured parasites.

parasite density (p/μL)	% detection (n/N)		
	one-step	exon1-nested	exon2-nested
200	100 (15/15)	100 (15/15)	86.7 (13/15)
20	100 (15/15)	100 (15/15)	73.3 (11/15)
2	46.7 (7/15)	80 (12/15)	60 (9/15)
0.2	0 (0/15)	6.7 (1/15)	0 (0/15)
0.02	0 (0/15)	0 (0/15)	0 (0/15)
0.002	0 (0/15)	0 (0/15)	0 (0/15)

#### Clinical isolates: Comparative sensitivity of nested and one-step methods

Sensitivity, specificity, PPV and NPV were compared to detection by PET PCR (Table 3). All methods had comparable sensitivities and specificities, with both nested PCRs having higher PPVs.

**Table 3:** Sensitivity, specificity, positive predictive value and negative predictive value of *pfhrp2* detecting PCR methods compared to PET PCR.

Method	*pfhrp2* result	PET PCR +	PET PCR-	total	Sensitivity	Specificity	PPV	NPV
					(95% CI)	(95% CI)	(95% CI)	(95% CI)
One-step PCR	+	95	1	116	95.0%	93.8%	99.0%	75.0%
	-	5	15		(88.7% -98.4%)	(69.8% - 99.8%)	(93.4%- 99.8%)	(55.9% - 87.7%)
Nested exon 1	+	99	0	116	99.0%	100.0%	100.0%	94.1%
	-	1	16		(94.6% - 99.9%)	(79.4% - 100.0%)		(69.5% - 99.1%)
Nested exon 2	+	98	0	116	98.0%	100.0%	100.0%	88.9%
	-	2	16		(93.0% - 99.8%)	(74.4% - 100.0%)		(67.0% - 96.9%)

Abbreviations are as follows, PPV = positive predictive value, NPV = negative predictive value, CI = confidence interval.

Detection by one-step PCR was then separately compared to detection by exon 1 and exon 2 nested PCRs for PET PCR positive samples only. There was no significant difference in sensitivity comparing one step and exon 1 PCR (p = 0.2137) or when comparing one step and exon 2 PCR (p = 0.4416), Prop test, performed using R version 3.5.0.

### Specificity against *pfhrp2*-deleted, and malaria negative samples

Twenty *P*. *falciparum* clinical isolates from Peru (not included in the assessment above) with previously confirmed *pfhrp2* deletion and an additional 14 malaria negative domestic surveillance samples (included in the above assessment) were combined to assess the specificity of each PCR method. All Peruvian samples were confirmed to be *P*. *falciparum* positive only by Snounou PCR, which was performed according to standard protocols outside of this study [45]. Mean parasite density was 13,716 p/μL (range: 976–55,560) which was determined by PET PCR. The 14 malaria negative domestic surveillance samples, were excluded as being malaria positive also using PET PCR, outside of this study. Results are outlined in S2 Table. Both one-step PCR and exon 2 PCR correctly classified 100% (34/34) samples as *pfhrp2* negative. In contrast, the exon 1 PCR misclassified two high density samples (parasite densities: 24,600 and 30,830 p/μL) as HRP2 exon 1 positive. These two PCR positive reactions were subjected to Sanger sequencing, and the amplicon confirmed to be *pfhrp3*.

### Practicality and cost of methods

Table 4 outlines a comparison of the one-step PCR and nested PCR protocols. The one-step PCR is 71.3% less expensive, and faster both in terms of hands on preparation time (80.0% less time) and thermocycling time (55.6% less time).

**Table 4:** Comparison of one-step and nested PCR protocol features.

Protocol attributes	One-step PCR	Nested PCRs
Cost (per sample)	$1.23	$4.28
Bench preparation time*	30 mins	2.5 hours
Thermocycling time	2 hours 40 mins	6 hours
Number of primers	2	8

* For preparing 96 samples

### One-step PCR detection based on parasite density

**Fig 2:**
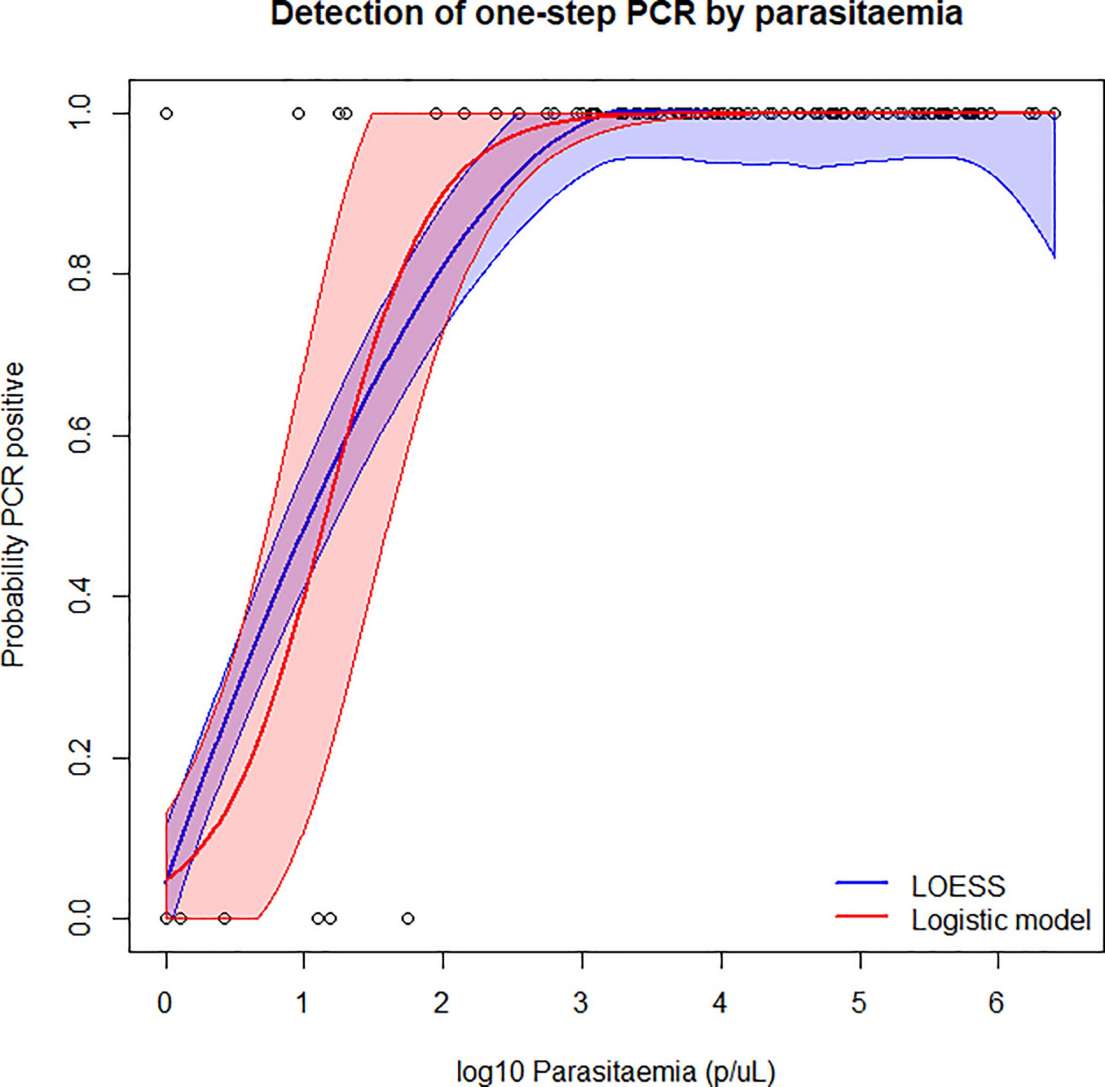
Probability of being one-step PCR positive, by log parasitaemia. 95% confidence intervals of the LOESS and logistic models are indicated by the color bands.

To further evaluate the new method, sensitivity of one-step PCR was assessed according to clinical isolate parasite density, Fig 2. 95% level of detection by one-step PCR was estimated at 198p/μL (95% CI: 24-674p/μL).

### One-step- PCR amplification from whole blood and dried blood samples

**Fig 3:**
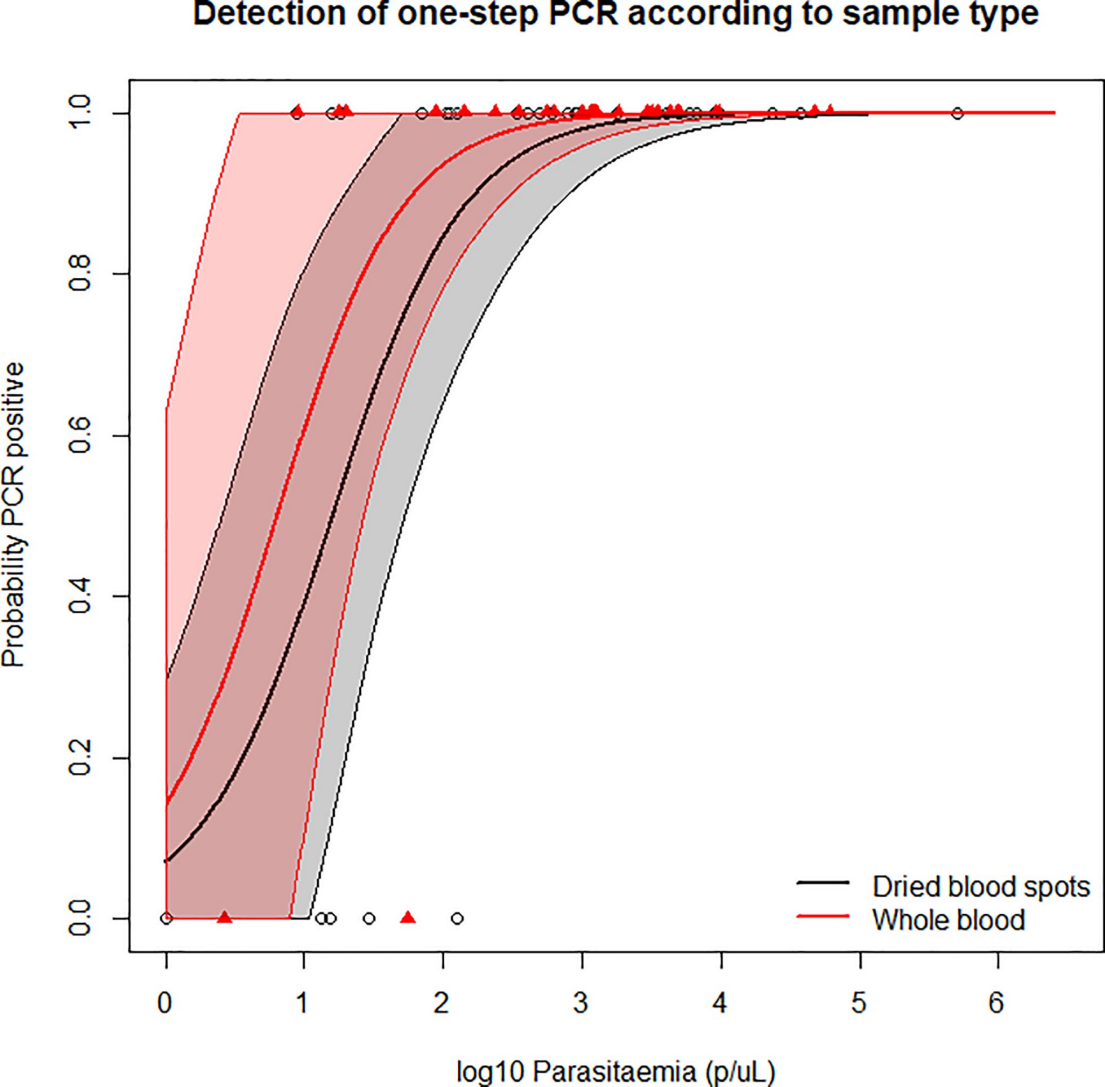
Detection of one-step PCR by log parasitaemia according to sample type. 95% confidence intervals are indicated by the color bands.

To determine if *pfhrp2* detection by one-step PCR was affected by sample collection type, detection from 30 WB (selected from the domestic surveillance samples) and 21 DBS samples from a health facility survey in Mozambique and 9 DBS samples from a TES in Angola [46], were compared, Fig 3. 95% level of detection by one-step PCR was estimated at 385p/μL (95% CI: 31-2133p/μL) for DBS, and 133p/μL (95% CI: 3-793p/μL) for WB samples.

### Application of the WHO *pfhrp2* deletion determination algorithm

The WHO algorithm for determination of *pfhrp2* deletion status was applied to 95 clinical samples (the rest of the stocks were depleted and could not have *pfmsp* genotyping performed) plus the 24 health facility survey samples from Mozambique, and 20 *pfhrp2*-deleted Peruvian samples, Table 5. It is important to note there is differential sensitivity between the three PCR methods when tested against titrated cultured isolates. The LOD, determined as the last 100% detected dilution was 200p/μL for *pfmsp-1*, 20p/μL for *pfmsp-2* (unpublished work performed outside of this study), and 20p/μL for the one-step PCR.

**Table 5:** *pfhrp2* deletion classification according to one-step *pfhrp2* PCR and single copy gene presence.

Interpretation	PCR result profile			Parasitaemia p/μL, mean (range)	Number of isolates
	One-step *pfhrp2* result	*pfmsp-1* result	*pfmsp-2* result		
Malaria negative	Negative	Negative	Negative	0 (0–1)	15
*Pfhrp2* deleted (correct)	Negative	Positive	Positive	13,716 (976–55,560)	20
(incorrect)	Negative	Positive	Positive	47.2 (3–125.9)	3
Indeterminate	Negative	Positive	Negative	n/a	0
	Negative	Negative	Positive	n/a	0
*Pfhrp2* positive	Positive	Positive	Positive	184,731 (9–2,559,000)	101
	Positive	Positive	Negative	n/a	0
	Positive	Negative	Positive	n/a	0
				Total:	139

Some samples summarized in Table 3 were exhausted and could not be included in this assessment. Parasitaemia was determined by PET PCR. *pfhrp2* deleted isolates were all confirmed to be deleted, outside of this study.

However, when tested against clinical isolates, the sensitivity of the methods seemed similar. Only samples with a parasitaemia of 0 or 1p/μL were deemed malaria negative using this algorithm and samples positive for all 3 methods, and therefore classified as *pfhrp2* positive, had a parasitaemia as low as 9p/μL. Three samples were incorrectly classified as *pfhrp2* deleted. These isolates were negative using one-step PCR, but confirmed to be positive for *pfhrp2* by both the nested exon 1 and nested exon 2 PCRs. The parasite densities of these samples were 3p/μL, 13.1p/μL and 125.9p/μL. No samples showed indeterminate status, which was defined by just one single copy gene being PCR positive and the other being negative.

### Sequencing

To ensure one-step PCR amplicon produced sufficient quality sequences to characterize HRP2 repeat patterns, a subset (n = 42, 7 of which were DBS) of samples were randomly selected for Sanger sequencing. Due to the length of our PCR amplicon (1333 b.p. total), alignment of the forward and reverse sequence traces did not result in complete overlap to cover the entire length of *pfhrp2* (~1064 b.p.). The average length with overlapping sequence from both the forward and reverse traces was 340 bp (range 98–670 bp).

Nucleotide sequences were translated, and all amino acid sequences were uploaded to NCBI (accession numbers MN584690-MN584731). Amino acid repeat compositions are shown in Table 6. No sequences had the amino acid region specific to *pfhrp3*, which further confirmed all amplicons were *pfhrp2*, not *pfhrp3*. Average amino acid length was 308.1 (range 224.0–340.0), and all isolates started with repeat type 1 and 2, contained types 6 and 7 and ended with repeat type 12.

**Table 6:** Amino acid repeat composition of analyzed samples.

	repeat type	Isolates containing the repeat (n = 42) (n/N)	average number of repeats per isolate (range)	stdev
1	AHHAHHVAD	100.00 (42/42)	2.76 (1.0–6.0)	1.34
2	AHHAHHAAD	100.00 (42/42)	12.31 (5.0–15.0)	2.12
3	AHHAHHAAY	80.95 (34/42)	1.19 (0.0–2.0)	0.74
4	AHH	26.19 (11/42)	0.43 (0.0–3.0)	0.83
5	AHHAHHASD	83.33 (35/42)	0.86 (0.0–2.0)	0.42
6	AHHATD	100.00 (42/42)	3.05 (1.0–6.0)	1.03
7	AHHAAD	100.00 (42/42)	6.31 (3.0–10.0)	1.76
8	AHHAAY	97.62 (41/42)	1.19 (0.0–2.0)	0.45
9	AAY	0.00 (0/42)	0.0 (0.0–0.0)	0.00
10	AHHAAAHHATD	85.71 (36/42)	1.36 (0.0–2.0)	0.73
11	AHN	0.00 (0/42)	0.0 (0.0–0.0)	0.00
12	AHHAAAHHEAATH	100.00 (42/42)	1.0 (1.0–1.0)	0.15
13	AHHASD	7.14 (3/42)	0.07 (0.0–1.0)	0.26
14	AHHAHHATD	11.90 (5/42)	0.19 (0.0–2.0)	0.55
30	AHHAVD (novel)	2.38 (1/42)	1.0 (1.0–1.0)	0.00
31	SHHAAY (novel)	2.38 (1/42)	1.0 (1.0–1.0)	0.00

Repeat types 15–29 are specific to *pfhrp3*, and were therefore not found in our *pfhrp2* sequences. Repeats type 2 and 7 are targeted by RDT monoclonal antibodies.

Two new repeat types were identified in our samples, type 30 appeared once in a Liberian isolate, and repeat type 31, appeared once in a Ugandan isolate.

Current research suggests that monoclonal antibodies employed by HRP2 RDTs detect epitopes on repeat types 2 and 7 [31, 47]. Among our isolates, each sample had an average of 12.3 (range 5.0–15.0) blocks of repeat type 2, and an average of 6.3 (range 3.0–10.0) blocks of repeat type 7. The majority of isolates, 69.0% (29/42) belonged to HRP2 type B, followed by 19.0% (8/42) with type C and 11.9% (5/42) with repeat type A (see S3 Table for full details).

Unique combinations of all 16 observed repeat type patterns were assigned arbitrary codes (S3 Table) to identify if any appeared more than once. The majority of isolates, 88.1% (37/42) were unique, appearing in just 1 isolate. Among those with shared sequence types, 3 identical isolates were from Tanzania, and two identical isolates originated from Ghana and Kenya (S3 Table). The number of samples analyzed per each country, along with the mean amino acid length and sequence variance is detailed in Table 7. There were insufficient numbers of sequences per country to allow robust intra-country comparisons.

**Table 7:** Number and length of amino acids found in samples grouped by country.

Country	no analyzed	mean amino acid length (range)	unique variants (%)	shared variants (%)
Mali	2	321.5 (320–323)	100	0
Ghana	1	300	0	100
Cote D'Ivoire	2	265.5 (224–307)	100	0
Liberia	4	310.75 (290–326)	100	0
Sierra Leone	3	316.67 (306–333)	100	0
Nigeria	3	315.67 (302–329)	100	0
Congo	1	334	100	0
Angola	8	303.38 (287–332)	100	0
Kenya	2	307.5 (300–315)	50	50
Tanzania	3	303 (303–303)	0	100
Uganda	3	324.67 (306–335)	100	0
Malawi	1	319	100	0
Africa (unspecified)	1	340	100	0
Haiti	1	330	100	0
India, Thailand, Cambodia	1	256	100	0
Unknown	6	303.67(238–332)	100	0

## Discussion

The ideal protocol for *pfhrp2* deletion monitoring would be rapid, affordable and accurate. Currently, the two exons of *pfhrp2* are detected by amplifying exon 1 (or exon 1 and exon 2 in separate reactions) using a nested PCR protocol that requires 4 PCR reactions per sample [36]. Assay parameters are important considerations particularly when working with a constrained budget, or when determining suitability for scale up in large studies. While nested PCR is advantageous since it offers high sensitivity, it is expensive, laborious and subject to risk of contamination [48]. Additionally, the exon 1 nested PCR is prone to spurious amplification of exon 1 of the paralogue *pfhrp3* due to primer similarity [29]. Reporting the presence or absence of just exon 1, or just exon 2 is commonplace, but this is not always informative enough [36, 40]. While exon 1 includes the signal peptide and could indicate if protein is expressed [49], exon 2 contains the highly variable region with repeat types 2 and 7 that are targeted by RDT monoclonal antibodies. Reporting on presence of both exons can therefore indicate if HRP2 will be expressed and therefore if that particular strain would be expected to be detected by RDTs. Reporting presence and variation of both exons is optimal, but currently hampered by laborious PCR protocols, and therefore is not always done [36].

Here we have presented a one-step PCR method that enables more rapid and accurate detection of the entire *pfhrp2* gene. We consider the one-step protocol to have several advantages compared to the nested protocol. Not only is the one-step method faster in terms of hands on preparation and thermocycling time but it is also approximately ¼ the cost of the nested protocol, $1.23 per sample compared to $4.28. The one-step protocol is also more efficient since it typically is performed just once in order to obtain the expected results for the controls, and any results for the test samples. In contrast, the nested PCRs are prone to failing and often need to be repeated, especially the exon 2 PCR since the nest 1 product has to be diluted 1/200 for use in the nest 2 PCR. It is important to note, these repeat experiments have not been factored into the time and cost estimates generated here for the nested PCRs. The estimates reflect a best-case scenario, but realistically the cost and times discussed here would be inflated. In summary, when considering these attributes, the nested PCR has low suitability for scale up to high throughput, compared to the one-step method.

The most concerning feature of the nested PCR protocol is the close similarity between the *pfhrp2* and *pfhrp3* exon 1 forward primers which differ by just 1 base pair for the nest 1 primers and 2 base pairs for the nest 2 primers, all 3 of which are located at the primer ends. In our hands, and elsewhere, this similarity has been recognized as sufficient to result in spurious amplification of *pfhrp3* when aiming to amplify *pfhrp2* [29]. Consequently, identity of the nested exon 1 PCR product needs to be confirmed by sequencing to state with confidence if the product really is *pfhrp2* and not *pfhrp3*. When using the one-step protocol, the presence of a band on a gel is conclusive enough to identify *pfhrp2*, and product does not need to be sequenced which reduces the overall cost and expands the usability of *pfhrp2* deletion investigation to laboratories that do not support sequencing technologies.

Many nested exon 2 protocols, including the one described here [12, 50], have a primer located in repeat type 12. However, repeat type 12 has been reported as being uncommon among strains in Senegal [13, 51] and should this absence become commonplace, these assays may not amplify *pfhrp2* exon 2, which could lead to inaccurate over-reporting of *pfhrp2* deletion prevalence. Our one-step PCR amplifies outside of the gene in a region of low genetic complexity, and therefore is expected to avoid amplification issues related to variability within the gene.

We want to highlight that our experiments were performed in a well-equipped laboratory under optimal conditions and the majority of these samples were stored as WB. The analytical sensitivity (100% detection) of our PCR against cultured isolates was found to be 20p/μL, which is comparable to the nested exon 1 PCR, but more sensitive than the exon 2 PCR. When the one-step protocol was tested against all clinical isolates combined, 95% detection was estimated at 198p/μL, which when classified by sample type was 133p/μL for WB samples, and 385p/μL for DBS. It has previously been reported that DNA stored on dried blood spot can suffer from fragmentation [52], which can impact downstream applications. This, along with possible insufficient filter paper drying or storage [53], could all contribute to lower sensitivity seen from DBS.

In line with WHO recommendations suggesting approaches to investigate *pfhrp2* deletion [28], our one-step PCR is intended for clinically relevant samples from symptomatic patients presenting to health care facilities, who are pf-pLDH RDT or microscopy positive, but HRP2 RDT negative [28]. Typically these infections would be expected to have fairly high parasitaemia. It is important that a conservative approach is adopted to avoid inaccurate reporting of *pfhrp2* deletion prevalence. The limits of detection of the chosen *pfhrp2*, and two single copy gene PCR methods all need to be considered when deciding which sample parasitaemia threshold can confidently be used to investigate *pfhrp2* deletion. Previous reports of *pfhrp2* deletion from Democratic Republic of Congo [19] were questioned for this reason [35]. In those reports, a real time PCR method was used to detect parasite presence, and conclude which samples were *pfhrp2*-deleted. Concerns were raised since the real time method was likely more sensitive than the traditional PCR method used to amplify *pfhrp2*, thus potentially resulting in over-reporting of *pfhp2* deletion. We reported the presence of *pfhrp2* in the context of two single copy genes, *pfmsp-1* and *pfmsp-2* and while this application could confirm *pfhrp2* presence in samples with a parasitaemia as low as 9p/μL, it also incorrectly deemed 3 samples as *pfhrp2* deleted that had parasite densities of 3.0 (WB), 13.1 (DBS) and 125.9p/μL (DBS), suggesting these parasite densities are around the limit of detection for this assay. We therefore echo the WHO recommendations that *pfhrp2* deletion should be investigated in samples of higher parasite density to avoid inaccurate over reporting of *pfhrp2* deletion prevalence, especially if DBS are being used. In our hands, we feel the one-step PCR method can be used to investigate *pfhrp2* deletion in WB samples with a parasite density of ≥133p/μL, and in DBS samples with a parasitaemia of ≥385p/μL. In line with good practice, we do recommend that investigators determine the LOD of this PCR method in their hands (and with the single copy genes they are using) prior to use with field isolates, to avoid inaccurate reporting of *pfhrp2* deletion.

Our study was designed to validate the new PCR method, rather than provide robust assessment of HRP2 type variance within different countries. Samples are typically characterized according to their *pfhrp2* type, which is determined by multiplying the number of repeat type 2 by the number of repeat type 7, as follows: type A (>100), B (50–99) and C (<50) [47]. Presently, the relevance of the variation observed with the types of repeats and how this translates to variation in RDT detection is unclear. HRP2 repeat patterns were explored among a subset of our samples. In line with previous research, repeat types 2 and 7 were most variable in terms of total numbers found in isolates [34, 47]. All our isolates contained repeat types 2 and 7, and while the lowest HRP2 type (C) was 25, the relevance of this has been disputed [34, 47] and there is no reason to suspect this isolate (or any others used in this study) would not be detected by an HRP2 RDT. Early research speculated HRP2 type impacted detection by HRP2 RDTs, with a count of <43 (HRP2 type C) reported as resulting in poor RDT detection in low parasitaemia infections below 250 p/μL [7]. However, this finding was derived from a handful of cultured isolates (n = 16), and uncertainty remains since subsequent studies exploring RDT detection sensitivity for larger numbers of field isolates collected across India (n = 769) and from the WHO Global Product Testing Specimen Bank (n = 100) did not support this theory [33, 47], with no correlation found between the number of repeats and RDT detection, even in low density (<200/μL) samples. Since our sample set was chosen from a bank of retrospectively collected samples, we did not have RDT detection data to help clarify this relationship. What can be seen among our sequenced samples is that extensive variation was observed, which is consistent with previous studies [7, 11, 31, 34, 47, 54]. The relevance of this diversity is yet to be elucidated. Within our sample set, three samples from travelers returning from Tanzania had identical *pfhrp2* repeat patterns, and a sample from a traveler to Ghana and one from a traveler to Kenya also matched. The relatedness of these strains would need to be disentangled by robust genotyping characterization methods exploring diversity in less variable targets, such as neutral microsatellites.

### Limitations

We recognize that our new PCR method would not indicate the size of the deleted region, if the deletion expands outside of our primer locations, to the flanking regions and beyond. To explore presence of flanking regions, separate PCR reactions would need to be performed, which are well described elsewhere [36]. While larger deletions of 20Kb have been found [40] they have not been widely reported. Due to the size of our amplicon, we did not have coverage of both forward and reverse strand for the whole gene. Since the aim of this study was to produce a method to enable identification of regions with larger deletions, rather than single nucleotide polymorphisms (SNPs), this approach was considered sufficient.

It is also important to note, that in this study we had relatively low number of samples with parasitaemia below 100p/μL (n = 9), and preserved as DBS (n = 30), that were used for our detection estimates. Having larger numbers of samples in these groups would increase the accuracy of our conclusions presented for these sample types.

In this study, we have only focused on improving detection of *pfhrp2*, which is the immediate WHO priority to inform RDT policy decisions. We recognize amplification of *pfhrp3* is currently performed using nested PCR and is constrained by the aforementioned disadvantages. Given cross reactivity of HRP3 on HRP2-detecting RDTs, it would be of value to update the methodology used for *pfhrp3* detection. However, this was outside the scope of this study.

This study presents a novel PCR and sequencing method that enables more rapid, cheaper and more accurate detection (and characterization) of the whole of *pfhrp2* compared to previous nested PCR methods. The method will support more efficient surveillance for *pfhrp2* deletions, an important goal for WHO’s Global Malaria Program.

## Nucleotide sequence accession numbers

Our nucleotide sequences have been deposited in NBCI under the following accession numbers: MN584690-MN584731.

## Supporting information

S1 Table One-step PCR mastermix preparation. (XLSX)

S2 Table HRP2 and HRP3 detection positivity using the one-step and nested protocols against a panel of *pfhrp2* deleted samples. (XLSX)

S3 Table HRP2 repeat composition of samples subjected to Sanger sequencing. (XLSX)
